# Therapeutic potentials of cell death inhibitors in rats with cardiac ischaemia/reperfusion injury

**DOI:** 10.1111/jcmm.17275

**Published:** 2022-03-21

**Authors:** Ying Luo, Nattayaporn Apaijai, Suchan Liao, Chayodom Maneechote, Titikorn Chunchai, Busarin Arunsak, Juthipong Benjanuwattra, Panat Yanpiset, Siriporn C. Chattipakorn, Nipon Chattipakorn

**Affiliations:** ^1^ 26682 Cardiac Electrophysiology Research and Training Center Faculty of Medicine Chiang Mai University Chiang Mai Thailand; ^2^ 26682 Center of Excellence in Cardiac Electrophysiology Research Chiang Mai University Chiang Mai Thailand; ^3^ 26682 Cardiac Electrophysiology Unit Department of Physiology Faculty of Medicine Chiang Mai University Chiang Mai Thailand; ^4^ 26682 Department of Oral Biology and Diagnostic Science Faculty of Dentistry Chiang Mai University Chiang Mai Thailand

**Keywords:** apoptosis, cardiac ischaemia/reperfusion injury, ferroptosis, mitochondria, necroptosis

## Abstract

Growing evidence demonstrated that cell death pathways including ferroptosis, apoptosis and necroptosis contribute to cardiac ischaemia/reperfusion (I/R) injury. We hypothesized that ferroptosis, apoptosis and necroptosis contribute differently to myocardial damage during acute cardiac I/R injury. Rats underwent cardiac I/R or sham operation. I/R‐operated rats were divided into 4 groups: vehicle, apoptosis (Z‐vad), ferroptosis (Fer‐1) and necroptosis (Nec‐1) inhibition. Rats in each cell death inhibitor group were subdivided into 3 different dose regimens: low, medium and high. Infarct size, left ventricular (LV) function, arrhythmias and molecular mechanism were investigated. Cardiac I/R caused myocardial infarction, LV dysfunction, arrhythmias, mitochondrial dysfunction, mitochondrial dynamic imbalance, inflammation, apoptosis and ferroptosis. Infarct size, LV dysfunction, mitochondrial dysfunction, apoptosis and ferroptosis were all reduced to a similar extent in rats treated with Z‐vad (low and medium doses) or Fer‐1 (medium and high doses). Fer‐1 treatment also reduced mitochondrial dynamic imbalance and inflammation. No evidence of necroptosis was found in association with acute I/R injury, therefore Nec‐1 treatment could not be assessed. Apoptosis and ferroptosis, not necroptosis, contributed to myocardial damage in acute I/R injury. Inhibitors of these 2 pathways provided effective cardioprotection in rats with I/R injury though modulation of mitochondrial function and attenuated apoptosis and ferroptosis.

## 1 INTRODUCTION

Acute myocardial infarction (AMI) is one of global leading causes of death even though the incidence of the disease has decreased.^1^ Reperfusion therapy is an effective treatment rapidly returning the blood supply to the ischaemic myocardial region.^2^, ^3^ Unfortunately, reperfusion therapy can also cause the ischaemic myocardium to suffer more injury due to oxidative damage, inflammation and cardiac mitochondrial impairment. This results in myocardial cell death and left ventricular (LV) dysfunction and is known collectively as ischaemia/reperfusion (I/R) injury.^4^, ^5^

Various forms of cell death are reportedly involved in cardiac I/R injury including apoptosis, necroptosis and ferroptosis, which occur in a regulated manner,^6^ all of which potentially contributing to the size of the final myocardial infarction.^7^ These three types of cell death are controlled by different mediators.^4^, ^8^, ^9^ Caspase 3, widely acknowledged as being associated with apoptosis, is activated following inflammation, oxidative stress and mitochondrial dysfunction.^10^ Necroptosis is induced by inflammatory stimulation from receptor‐interacting protein kinase 1 (RIPK1), RIPK3 and mixed‐lineage kinase domain‐like protein (MLKL).^11^ Ferroptosis is regulated cell death initiated by reactive oxygen species (ROS), which are produced as a result of intracellular iron accumulation or extensive lipid peroxidation.^12^ An increase in acyl‐coA synthetase long‐chain family member 4 (ACSL4) provokes phospholipid oxidation via the enhancement of polyunsaturated fatty acid content, which together with a decrease in glutathione peroxidase 4 (GPX4) level, also results in ferroptosis.^11^ Mitochondrial dysfunction has also been shown to contribute to cell death during cardiac I/R injury via ROS overproduction, mitochondrial cytochrome *c* (Cyt *c*) leakage and the collapse of mitochondrial membrane potential.^13^

Many regulated cell death inhibitors exhibit cardioprotective effects against cardiac I/R injury.^14^, ^15^, ^16^ Z‐VAD.fmk (Z‐vad) is a pan‐caspase inhibitor, which attaches to the catalytic site of caspase proteases and decreases caspase 3 levels.^14^ Necrostatin‐1 (Nec‐1) diminishes RIPK1, RIPK3 and necroptosome (RIPK1‐RIPK3‐MLKL) formation.^15^ Ferrostatin‐1 (Fer‐1) is a synthetic antioxidant that specifically inhibits ROS associated lipid peroxide accumulation.^16^ Although these regulated cell death inhibitors have previously been shown to effectively limit myocardial infarct size and LV dysfunction,^14^, ^15^, ^16^ the comparative effects of these regulated cell death inhibitors in the same cardiac I/R setting have never been investigated. Understanding the contribution of each of these cell death pathways may open a new window of opportunity to inform and improve therapeutic approaches against cardiac I/R injury. Thus, we hypothesized that ferroptosis, apoptosis and necroptosis contribute differently to myocardial damage during acute cardiac I/R injury, and that inhibition of apoptosis, necroptosis and ferroptosis attenuates cardiac I/R injury at different degrees in rats via the reduction of oxidative stress, inflammation and mitochondrial dysfunction.

## 2 METHODS

### 2.1 Animals and experimental design

All animal procedures in this study adhered to the Guide for the Care and Use of Laboratory Animals (NIH Publications No. 8023, revised 1978) and ARRIVE guidelines for reporting animal research. The study protocol was approved by the Institutional Animal Care and Use Committee of the Faculty of Medicine, Chiang Mai University, Thailand (Permit no. 25/2563).

Male Wistar rats (400–500 g, *n *= 126) were purchased from the Nomura Siam International Co, Ltd. (Bangkok, Thailand) and housed in a temperature‐controlled room, with a 12‐hour light‐dark cycle. The rats were fed with a standard diet (CP082, Bangkok, Thailand) and water *ad libitum*. After one week of acclimatization, rats were randomly assigned into two operating groups: sham operation (*n *= 6) and cardiac I/R operation (*n *= 120). In the cardiac I/R group, rats received different treatments, specifically: (1) vehicle (10% DMSO in normal saline solution), (2) apoptosis inhibitor (Z‐vad, MyBioSource, USA) at low (1.65 mg/kg), medium (3.3 mg/kg) and high dosage (6.6 mg/kg), (3) necroptosis inhibitor (Nec‐1, Santa Cruz, USA) at low (1.65 mg/kg), medium (3.3 mg/kg) and high dose (6.6 mg/kg) and (4) ferroptosis inhibitor (Fer‐1, Sigma, USA) at low (1 mg/kg), medium (2 mg/kg) and high dose (4 mg/kg) via intravenous injection. Z‐vad, Fer‐1 and Nec‐1 are specific inhibitors for apoptosis, ferroptosis and necroptosis in cardiac I/R injury model, respectively. The concentration of chosen based on the data from previous studies. Z‐vad (3.3 mg/kg) and Fer‐1 (2.2 mg/kg) exerted cardioprotective effects against cardiac I/R injury in rats.^17^, ^18^ Therefore, 3.3 mg/kg of Z‐vad and 2 mg/kg of Fer‐1 were used as a medium dose in this study. The pharmacological responses of these agents were also tested during cardiac I/R injury in both lower and higher doses. Although 1.65 mg/kg of Nec‐1 was reported to reduce the infarct size in mice,^19^ it failed to decrease the infarct size in adult female rats.^20^ Due to these controversial findings on Nec‐1, we used 3.3 mg/kg of Nec‐1 as a medium dose, whereas 1.65 mg/kg was used as a low dose and 6.6 mg/kg as a high dose.

**FIGURE 1:**
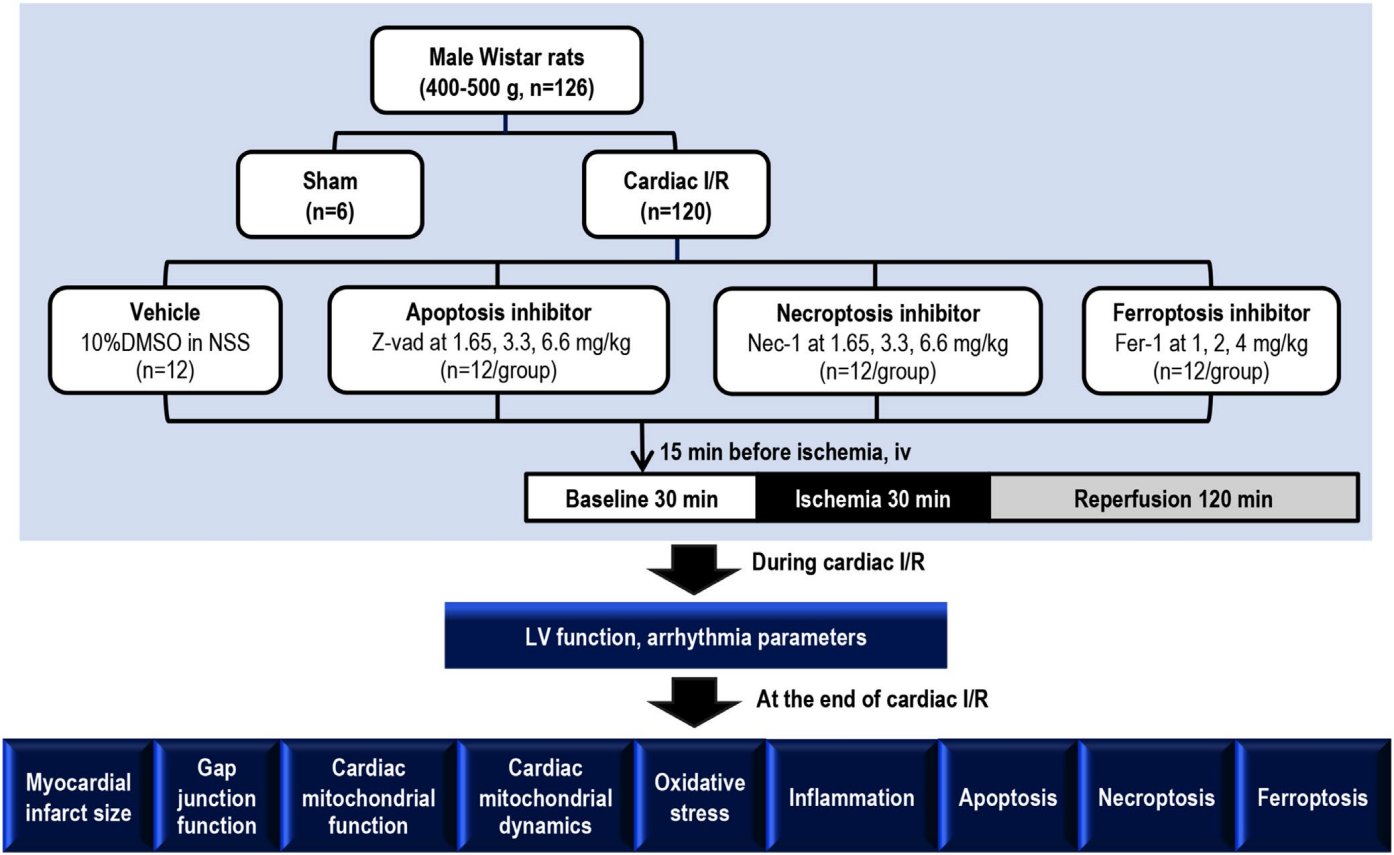
Diagram of the experimental protocol. Fer‐1: ferrostatin‐1; LV: left ventricular; Nec‐1: necrostatin‐1; I/R: ischaemia/reperfusion; DMSO: Dimethylsulfoxide; NSS: normal saline solution; Z‐vad: Z‐vad.fmk

Z‐vad, Nec‐1 and Fer‐1 were dissolved in 10% DMSO with normal saline solution. This concentration of DMSO has been shown to have no toxic effect on rats.^21^ Each regulated cell death inhibitor was given to the rats 15 min before the cardiac I/R operation. For the cardiac I/R protocol, myocardial ischaemia was initiated by ligation of the left anterior descending (LAD) coronary artery for 30 min, followed by reperfusion for 120 min.^22^, ^23^ Sham‐operated rats underwent a similar surgical procedure without LAD ligation. LV function was determined and lead II electrocardiogram was recorded to analyse arrhythmias throughout the cardiac I/R protocol. At the end of reperfusion, all rats were euthanized, and the heart was rapidly excised to determine infarct size (*n *= 6/group) and enable molecular studies (*n *= 6/group). A schematic diagram illustrating the experimental protocol is shown in Figure 1.

### 2.2 Cardiac I/R protocol

Rats were anaesthetized using Xylazine (0.15 mg/kg, LBS labs, Bangkok, Thailand) and Zoletil (50 mg/kg, Virbac Laboratories, France) via intramuscular injection. The rats were ventilated using a positive pressure ventilator (Cwe, Inc., USA). A thoracotomy was performed at the fourth intercostal space to expose the heart. LAD ligation was performed approximately 2 mm distal to its origin. Myocardial ischaemia was done by ligating the LAD for 30 min, followed by 120 min of reperfusion. Elevation of the ST segment on lead II electrocardiogram was considered a successful myocardial ischaemia.^22^

### 2.3 Determination of arrhythmias

The lead II electrocardiogram was recorded throughout the I/R protocol to analyse cardiac arrhythmia parameters including QRS duration, QTc interval, time to the first onset of ventricular tachycardia (VT)/ventricular fibrillation (VF) and arrhythmia score. Quantification of arrhythmia score was followed using the guidelines as described by Curtis and Walker.^24^

### 2.4 Determination of LV function

The LV function was recorded throughout the cardiac I/R operation using an invasive pressure‐volume (P‐V) loop system (Transonic Scisence, Canada). The P‐V catheter was inserted into the LV chamber via the right carotid artery before cardiac I/R surgery. LV function parameters including Heart rate (HR), LV end‐systolic pressure (LVESP), LV end‐diastolic pressure (LVEDP), d*P*/d*t* max (+d*P*/d*t*), d*P*/d*t* min (−d*P*/d*t*), stroke volume (SV) and %LV ejection fraction (%LVEF) were determined. The P‐V loop data were collected and analysed by LabScribe 2 program (Iworx, USA) at the baseline, the end of the ischaemia and the end of the reperfusion.^25^

### 2.5 Determination of myocardial infarct size

At the end of the cardiac I/R operation, the LAD was re‐occluded, and the heart was excised under deep anaesthesia. The heart was perfused with 1 ml of 0.5% Evans‐blue dye in normal saline solution via the aorta. Then, it was frozen at −20°C overnight and then sectioned horizontally from the apex to the occlusion site. The heart slices were incubated with 1% Triphenyl tetrazolium chloride (TTC, Sigma, USA) solution in phosphate buffer saline for 12 min at 37°C. The blue area indicated an area which was not supplied by the LAD. The area with viable tissue was shown in red, while the infarct area was established in white. The area at risk (AAR) and infarct size/AAR were analysed using Image J software (NIH, USA).^22^

### 2.6 Determination of cardiac mitochondrial function

At the end of the cardiac I/R surgery, the heart was dissected and separated into ischaemic (I) and remote (R) areas. The heart tissue was homogenized, and cardiac mitochondria were obtained by differential centrifugation.^22^, ^26^ Bicinchoninic acid assay was used (Sigma, USA) to determine mitochondrial protein concentration. 0.4 mg/ml of mitochondrial protein was used for mitochondrial function assays, including mitochondrial ROS production, mitochondrial membrane potential changes and swelling.

To assess mitochondrial ROS production, cardiac mitochondria were stained with 2 μM of dichloro‐dihydro‐fluorescein diacetate dye (DCFH‐DA, Sigma, USA) and incubated at room temperature for 20 min. The ROS level was measured using a fluorescent microplate reader (excitation wavelength 485 nm and emission wavelength 530 nm, BioTek Instruments, USA).^22^

For mitochondrial membrane potential changes, mitochondria were stained with 310 nM of JC‐1 dye (Thermo Fisher Scientific, USA), and incubated at 37°C for 30 min. JC‐1 fluorescence was measured using a fluorescent microplate reader (excitation wavelength 485 nm and emission wavelength 530/590 nm, BioTek Instruments, USA). A decrease in red/green fluorescence ratio indicates mitochondrial membrane depolarization.^22^

Mitochondrial swelling was measured by a change in optical density of mitochondrial suspension using a microplate reader, and the morphology of mitochondria was visualized by transmission electron microscope (Jeol Ltd., Japan).^23^

### 2.7 Western blot analysis

Analysis of the expression of proteins associated with apoptosis, necroptosis, ferroptosis, inflammation and cardiac gap junction was performed in cardiac tissue from both I and R areas. In addition, isolated mitochondria from I and R areas were used to investigate the expression of proteins associated with cardiac mitochondrial dynamics. Total protein from cardiac tissue (50–80 mg/ml) and mitochondrial fragment (0.4 mg/ml) were mixed with a loading buffer. The protein was heated at 95°C for 10 min and loaded onto either a 10% or 12.5% gradient SDS‐polyacrylamide gel. Then, they were transferred to a nitrocellulose membrane in a glycine/methanol transfer buffer in a wet tank transfer system (Bio‐Rad Laboratories, Inc., USA). The nitrocellulose membranes were incubated with either 5% bovine serum albumin (BSA) or skim milk in tris‐buffered saline with 0.1% of Tween 20 (TBST) buffer for 1 hour at room temperature. Then, they were incubated with the following primary antibodies at 4 °C for 16 hours: apoptosis markers (Bax, Bcl‐2, Cyt *c*, Caspase 3), necroptosis markers (Caspase 8, RIPK1, p‐RIPK1^Ser166^, RIPK3, p‐RIPK3^Ser232^, MLKL, p‐MLKL), ferroptosis markers (ACSL4, GPX4), antioxidant marker (SOD2); inflammatory markers (TNF‐α, NF‐κB, p‐NF‐κB^Ser536^), cardiac gap junction markers (Cx43, p‐Cx43^Ser368^), and mitochondrial dynamics markers (Drp1, p‐Drp1^Ser616^, Mfn1, Mfn2, OPA1). The following loading controls were used in this study: GAPDH and VDAC. The membranes were washed in TBST buffer, and the bound antibodies were detected through anti‐mouse or anti‐rabbit IgG conjugated with horseradish peroxidase (HRP). The western blot was developed by enhanced chemiluminescence assay (Bio‐Rad Laboratories, Inc., USA), and the pictures was taken using the ChemiDoc touching system (Bio‐Rad Laboratories, Inc., USA). Data were analysed using Image J software (NIH, USA).^23^

### 2.8 Terminal deoxynucleotidyl transferase dUTP nick end labelling (TUNEL) assays

In this study, TUNEL positive cells were used to give representative pictures of apoptotic cells. The ischaemic heart was sliced into 10 μm thick slices in a cryostat (Leica, Germany) and incubated with proteinase k solution followed by the enzyme terminal deoxynucleotidyl transferase incorporation of EdUTP into dsDNA strand breaks. TUNEL positive cells were detected at the excitation wavelength of 650 nm and emission wavelength of 670 nm using a fluorescent microscope (Nikon, Japan). The nucleus was stained with 4′,6‐diamidino‐2‐phenylindole (DAPI) and detected at the excitation wavelength of 358 nm and the emission wavelength of 461 nm.^27^

### 2.9 Statistical analysis

The data for each experiment was presented as the mean ± standard error of the mean (SEM). The data pertinent to mitochondrial function and western blots were represented as the ratio of ischaemic/remote value. Data were analysed using GraphPad Prism 9.0 software (GraphPad Software, USA). The differences between groups were analysed using a one‐way or two‐way ANOVA followed by an LSD post hoc test, unpaired Student's *t*‐test or non‐parametric test. A value of *p* < 0.05 was considered a statistically significant.

## 3 RESULTS

### 3.1 Apoptosis and ferroptosis, but not necroptosis, involved in the pathogenesis of cardiac I/R injury

**FIGURE 2:**
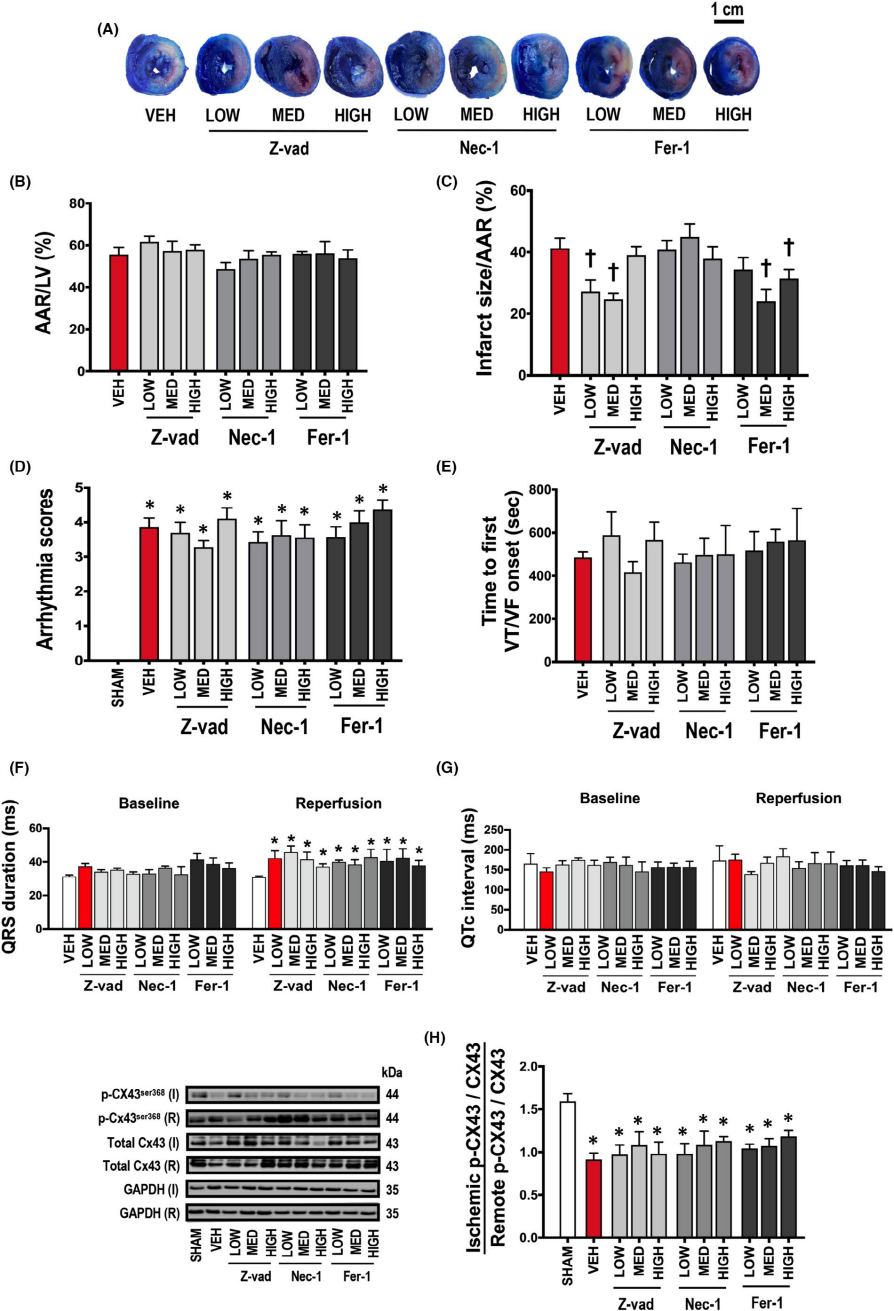
Effects of apoptosis, necroptosis and ferroptosis inhibitors on myocardial infarction and arrhythmias in rats with cardiac I/R injury (*n *= 6/group). (A) Representative pictures of myocardial infarct size from Evans‐blue & TTC staining (scale bar = 1 cm), (B) Quantitative analysis of %AAR/LV area, (C) Quantitative analysis of % myocardial infarct size/AAR, (D) Arrhythmia score, (E) Time to the first VT/VF onset; (F) QRS duration, (G) QTc interval, (H) The ratio of p‐Cx43^Ser368^/total Cx43 expression. Data are presented as means ± SEM. *p < 0.05 vs sham, †*p* < 0.05 vs vehicle. AAR: area at risk; Cx43: connexin 43; Fer‐1: ferrostatin‐1; GAPDH: glyceraldehyde 3‐phosphate dehydrogenase; HIGH: high dose; LOW: low dose; LV: left ventricular; MED: medium dose; Nec‐1: necrostatin‐1; p‐Cx43^ser368^: phosphorylation of connexin43 at serine‐368; VEH: vehicle; VF: ventricular fibrillation; VT: ventricular tachycardia; Z‐vad: Z‐vad.fmk

**FIGURE 3:**
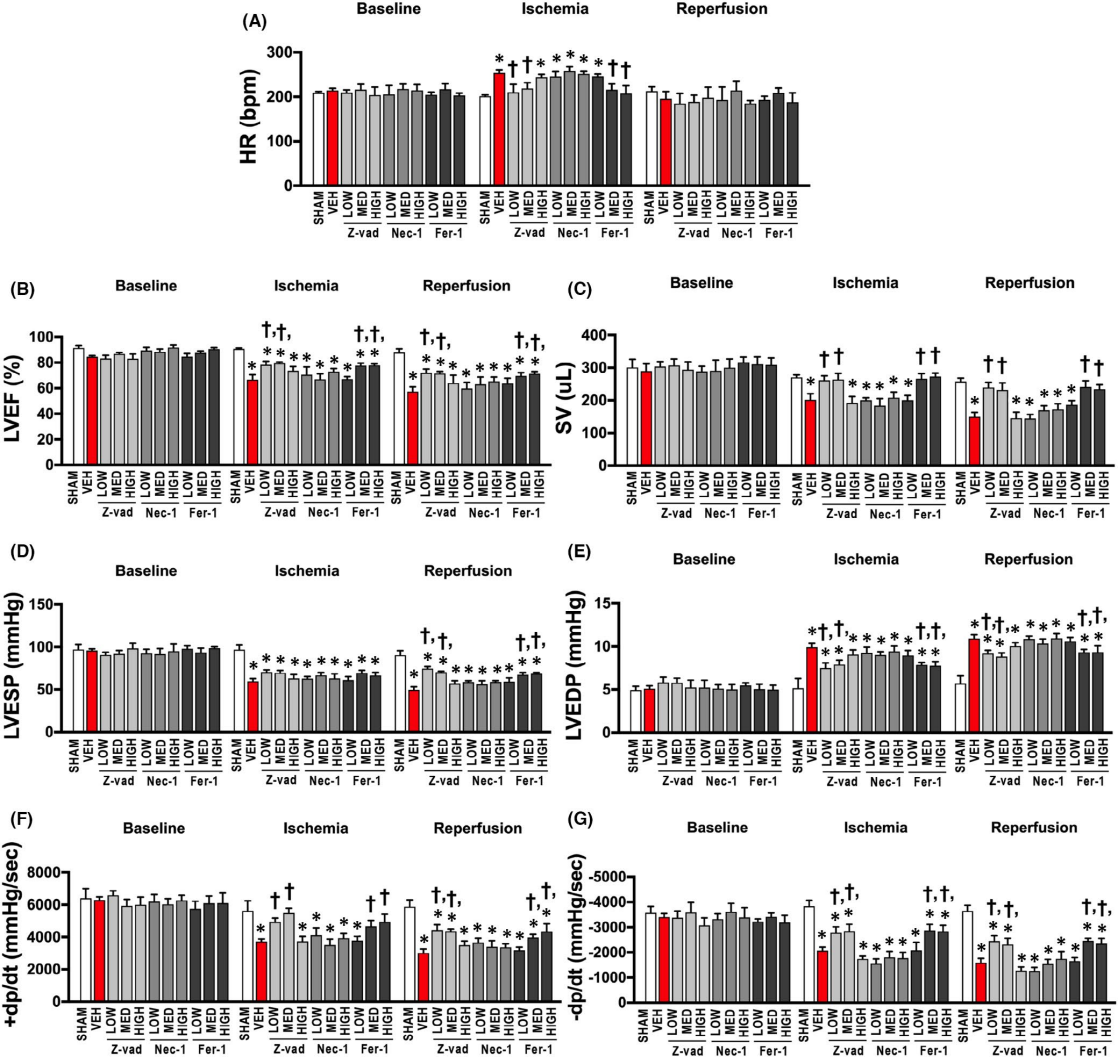
Effects of apoptosis, necroptosis and ferroptosis inhibitors on LV function in rats with cardiac I/R injury (*n *= 6/group). (A) HR, (B) %LVEF, (C) SV, (D) LVESP, (E) LVEDP, (F) +d*P*/d*t*, (G) −d*P*/d*t*. Data are presented as means ± SEM. *p < 0.05 vs sham, †*p* < 0.05 vs vehicle. +d*P*/d*t*: maximum rate of rise of left ventricular pressure; −d*P*/d*t*: minimum rate of rise of left ventricular pressure; Fer‐1: ferrostatin‐1; HIGH: high dose; HR: heart rate; LOW: low dose; LVEDP: left ventricular end‐diastolic pressure; LVEF: left ventricular ejection fraction; LVESP: left ventricular end‐systolic pressure; MED: medium dose; Nec‐1: necrostatin‐1; SV: stroke volume; VEH: vehicle; Z‐vad: Z‐vad.fmk

**FIGURE 4:**
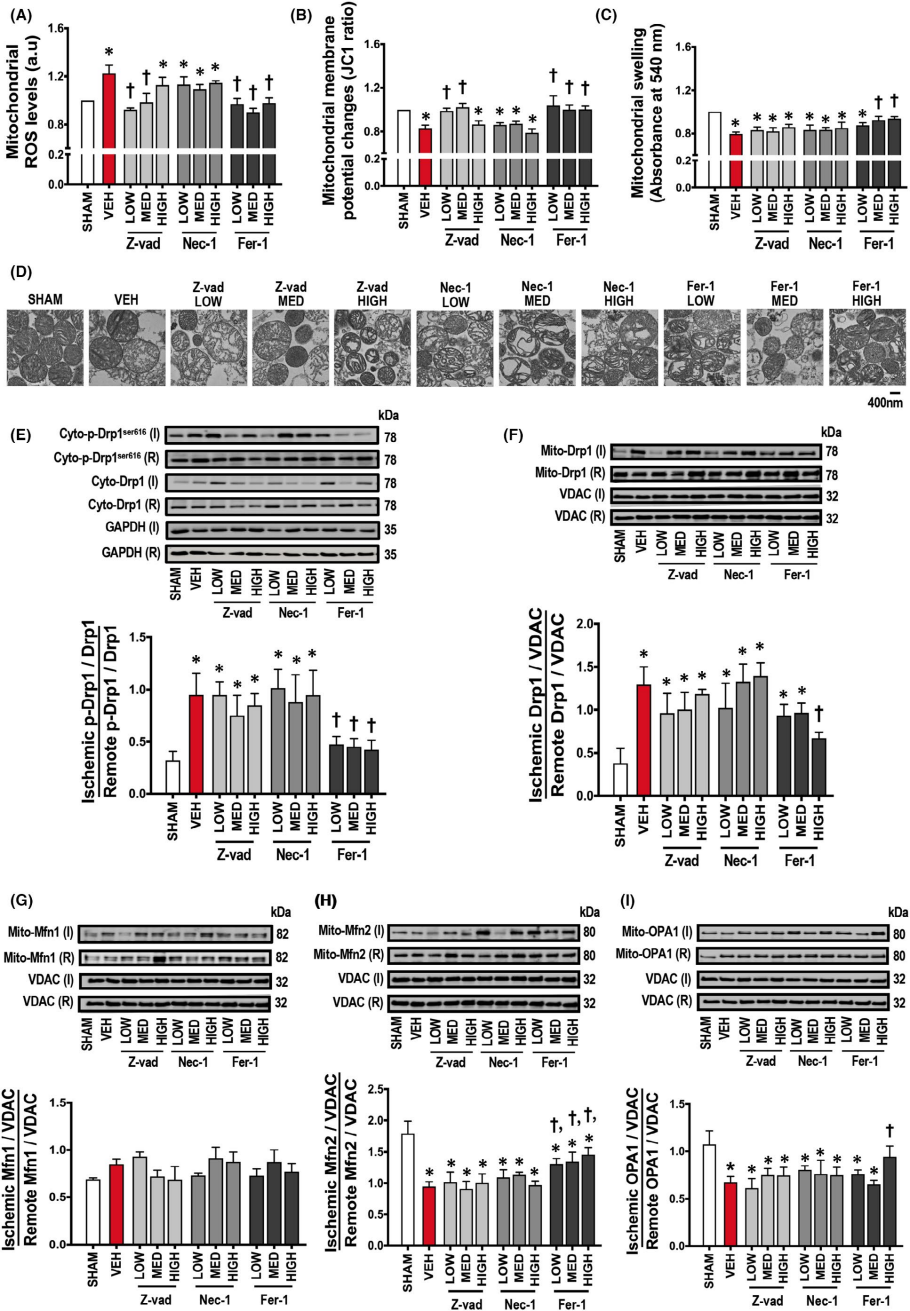
Effects of apoptosis, necroptosis and ferroptosis inhibitors on cardiac mitochondrial function and mitochondrial dynamics in rats with cardiac I/R injury (*n *= 6/group). (A) Mitochondrial ROS levels, (B) Mitochondrial membrane potential changes, (C) Mitochondrial swelling, (D) Representative TEM pictures of mitochondria (scale bar = 400 nm, (E) Cytosolic p‐Drp1^ser616^/Drp1, (F) Mitochondrial Drp1/VDAC, (G) Mitochondrial Mfn1/VDAC, (H) Mitochondrial Mfn2/VDAC, (I) Mitochondrial OPA1/VDAC. Data are presented as means ± SEM. *p < 0.05 vs sham, †*p* < 0.05 vs vehicle. Drp1: dynamin related protein 1; Fer‐1: ferrostatin‐1; GAPDH: glyceraldehyde 3‐phosphate dehydrogenase; HIGH: high dose; I: ischaemic; LOW: low dose; MED: medium dose; Mfn: mitofusin; Nec‐1: necrostatin‐1; OPA: optic atrophy protein; p: phosphorylation; R: remote; ROS: reactive oxygen species; TEM: transmission electron microscopy; VDAC: voltage‐dependent anion channel; VEH: vehicle; Z‐vad: Z‐vad.fmk

**FIGURE 5:**
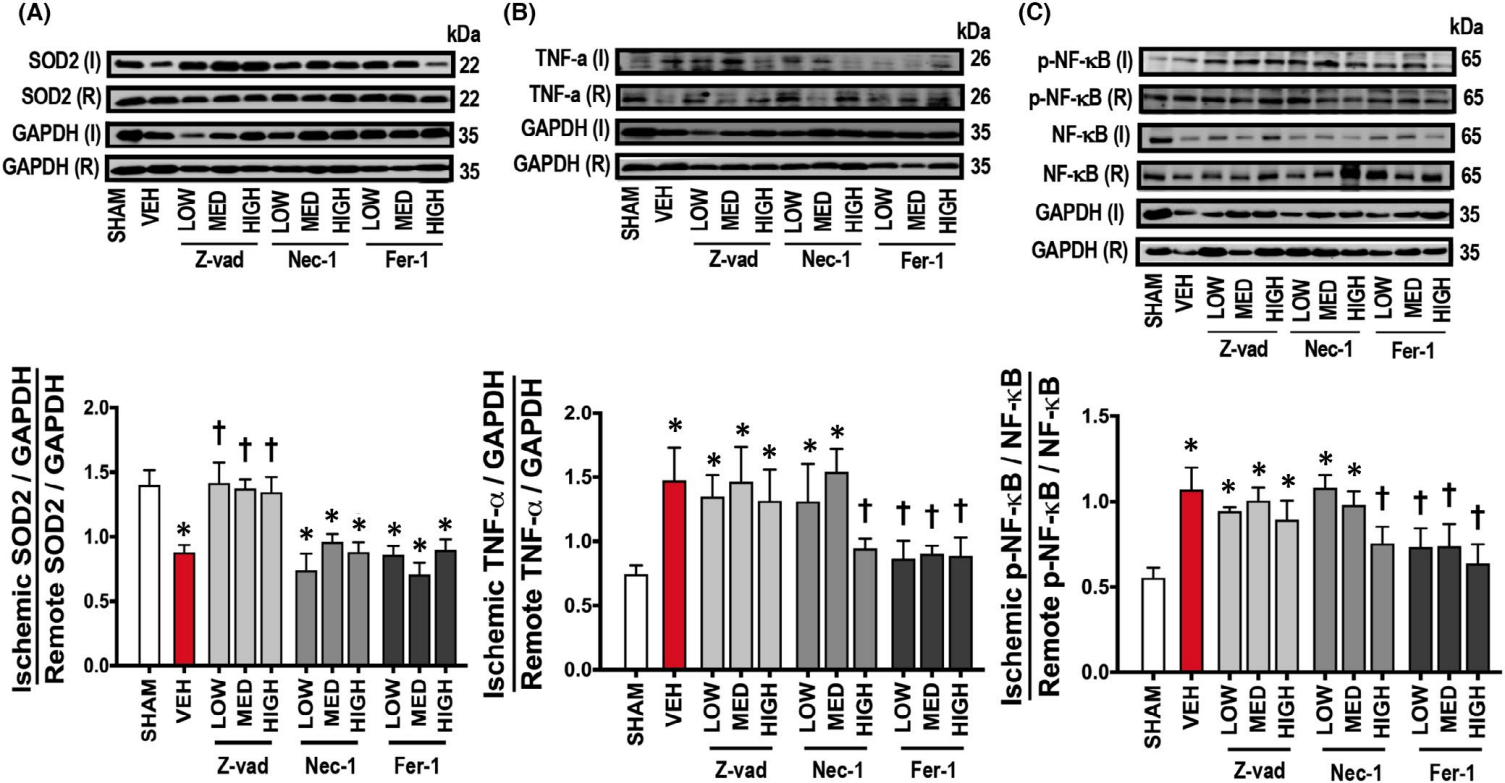
Effects of apoptosis, necroptosis and ferroptosis inhibitors on cardiac antioxidant and inflammation in rats with cardiac I/R injury (*n *= 6/group). (A) SOD2, (B) TNF‐α, (C) p‐NF‐κB/NF‐κB. Data are presented as means ± SEM. *p < 0.05 vs sham, †*p* < 0.05 vs vehicle. Fer‐1: ferrostatin‐1; GAPDH: glyceraldehyde 3‐phosphate dehydrogenase; HIGH: high dose; I: ischaemic; LOW: low dose; MED: medium dose; Nec‐1: necrostatin‐1; NF‐κB: nuclear factor kappa B; p: phosphorylation; R: remote; SOD: superoxide dismutase; TNF‐α: tumour necrosis factor alpha; VEH: vehicle; Z‐vad: Z‐vad.fmk

Cardiac I/R injury led to myocardial infarction, as shown in the representative pictures in Figure 2A. LV dysfunction together with cardiac arrhythmia as indicated by increased arrhythmia scores were observed following cardiac I/R injury (Figure 2D), compared to sham‐operated rats. LV function was not altered in the sham group at any point (Figure 3A‐F). During cardiac I/R, vehicle‐treated rats exhibited LV dysfunction as indicated by decreased %LVEF, SV, LVESP, ±d*P*/d*t* and increased HR, LVEDP in both ischaemic and reperfusion periods, compared to sham‐operated rats (Figure 3A‐G). Mechanistically, cardiac I/R injury caused mitochondrial dysfunction, indicated by increased mitochondrial ROS levels, mitochondrial membrane depolarization, and mitochondrial swelling, compared to sham‐operated rats (Figure 4A‐D). In addition, an imbalance of cardiac mitochondrial dynamics was observed in rats with I/R injury, as indicated by increased mitochondrial fission proteins (cytosolic p‐Drp1^ser616^/Drp1 and mitochondrial Drp1 protein levels) and decreased mitochondrial fusion markers (mitochondrial Mfn2 and OPA1 protein levels), compared to sham‐operated rats (Figure 4E, F, H, I). Mitochondrial antioxidant was suppressed, and cardiac inflammation was induced in rats with I/R injury as shown by a decrease in the SOD2 protein levels, and an increase in the TNF‐α and p‐NF‐κB/NF‐κB protein levels, compared to sham‐operated rats (Figure 5A‐C).

**FIGURE 6:**
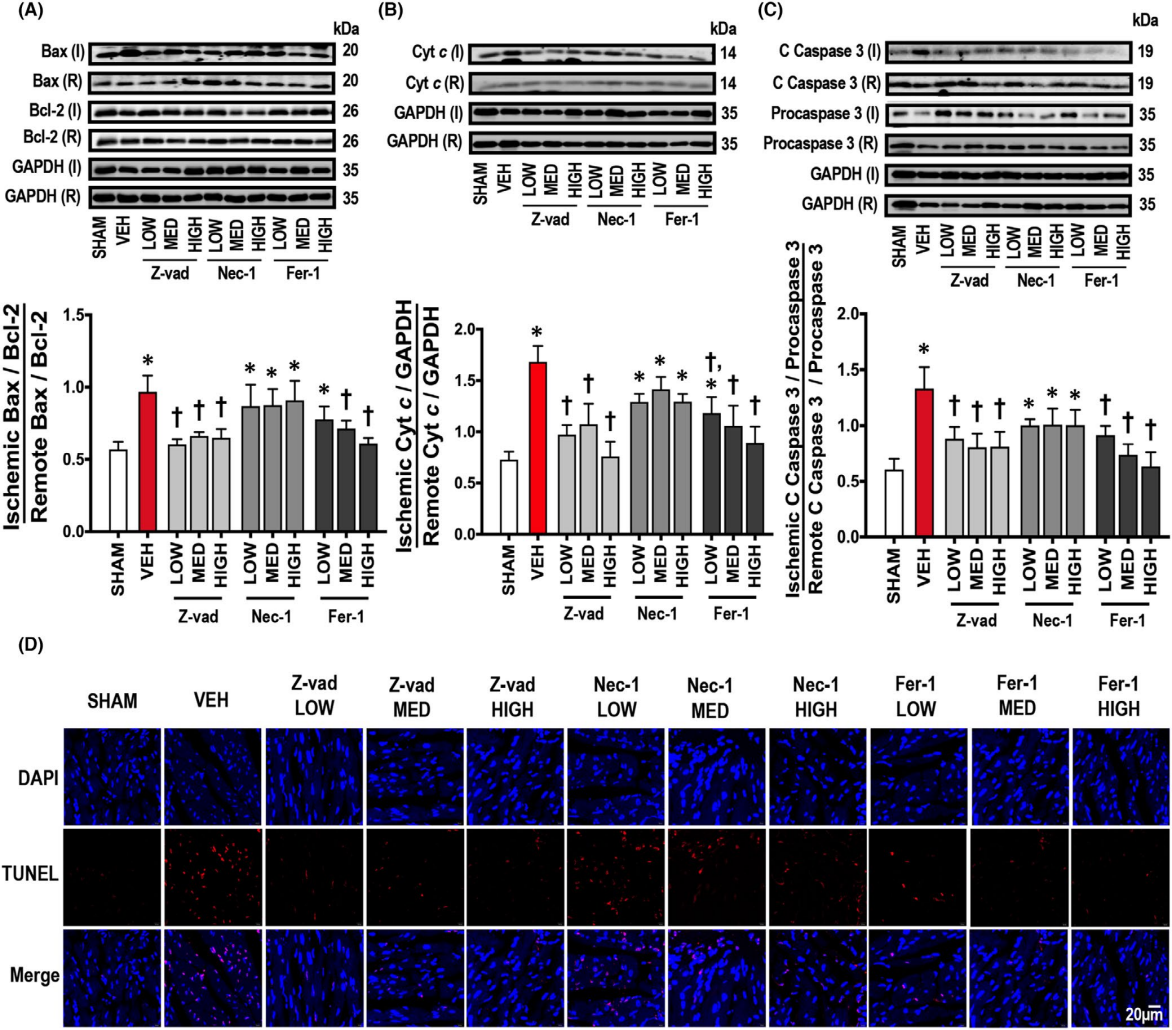
Effects of apoptosis, necroptosis and ferroptosis inhibitors on cardiac apoptosis in rats with cardiac I/R injury (*n* = 6/group). (A) Bax/Bcl‐2, (B) Cyt *c*, (C) Cleaved caspase 3/Procaspase 3, (D) Representative TUNEL pictures (scale bar = 20 μm). Data are presented as means ± SEM. *p < 0.05 vs sham, †*p* < 0.05 vs vehicle. Bax: Bcl‐2 Associated X; C: cleaved; Cyt *c*: cytochrome c; DAPI: 4′,6‐diamidino‐2‐phenylindole; Fer‐1: ferrostatin‐1; GAPDH: glyceraldehyde 3‐phosphate dehydrogenase; HIGH: high dose; I: ischaemic; LOW: low dose; MED: medium dose; Nec‐1: necrostatin‐1; R: remote; TUNEL: Terminal deoxynucleotidyl transferase dUTP nick end labelling; VEH: vehicle; Z‐vad: Z‐vad.fmk

**FIGURE 7:**
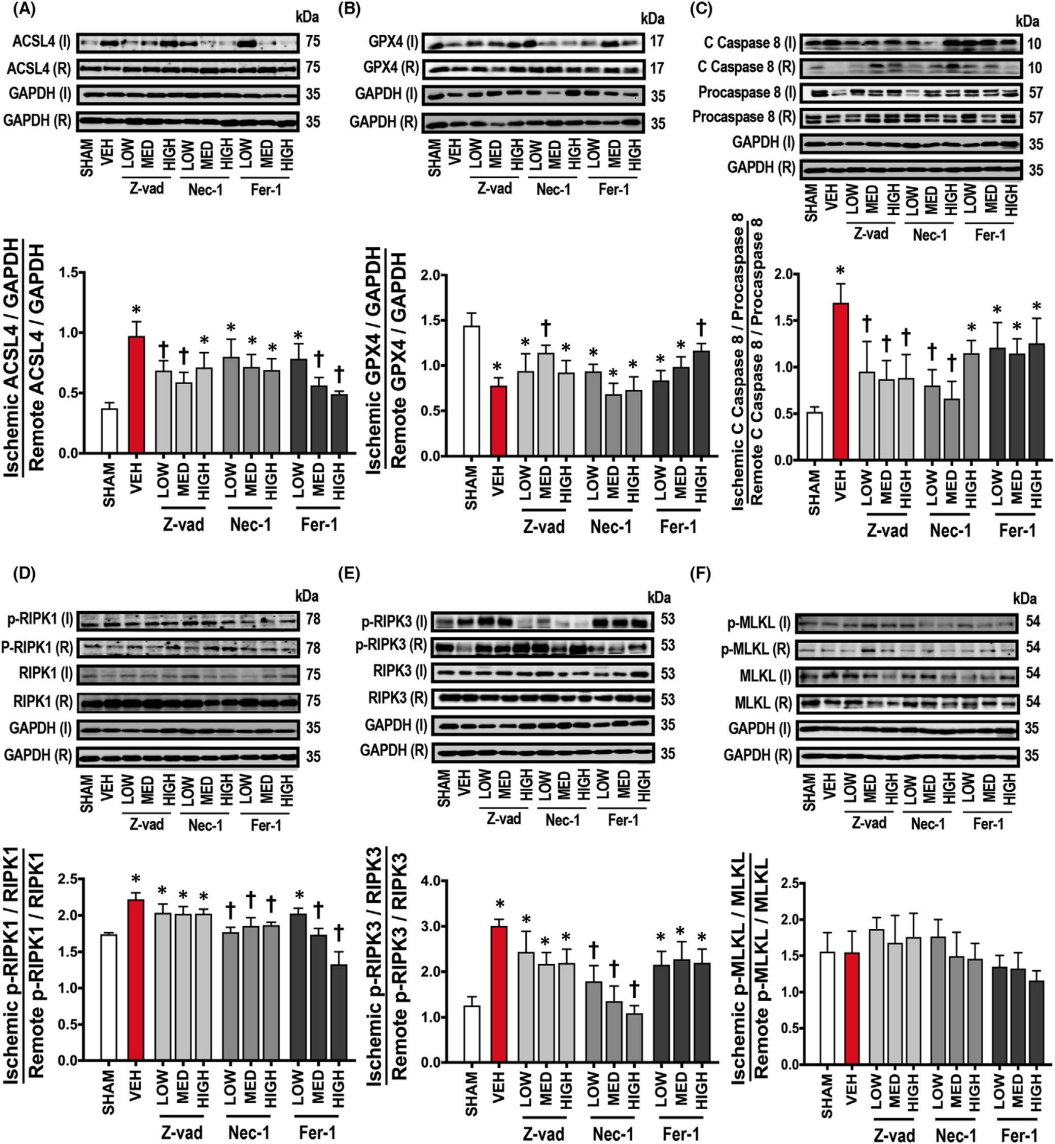
Effects of apoptosis, necroptosis and ferroptosis inhibitors on cardiac ferroptosis and necroptosis in rats with cardiac I/R injury (*n *= 6/group). (A) ACSL4, (B) GPX4, (C) Cleaved caspase 8/Procaspase 8, (D) p‐RIPK1/RIPK1, (E) p‐RIPK3/RIPK3, (F) p‐MLKL/MLKL. Data are presented as means ± SEM. *p < 0.05 vs sham, †*p* < 0.05 vs vehicle. ACSL4: acyl‐CoA synthetase long‐chain family member 4; C: cleaved; Fer‐1: ferrostatin‐1; GAPDH: glyceraldehyde 3‐phosphate dehydrogenase; GPX4: glutathione peroxidase 4; HIGH: high dose; I: ischaemic; LOW: low dose; MED: medium dose; MLKL: mixed‐lineage kinase domain‐like protein; Nec‐1: necrostatin‐1; p: phosphorylation; R: remote; RIPK: Receptor‐interacting serine/threonine‐protein kinase; VEH: vehicle; Z‐vad: Z‐vad.fmk

Importantly, the involvement of different cell death pathways in cardiac I/R injury was investigated in this study. Our results demonstrated that cardiac I/R injury led to myocardial apoptosis, as indicated by an increased Bax/Bcl‐2 ratio, Cyt *c* and cleaved caspase 3/Procaspase 3 protein levels, compared to sham‐operated rats (Figure 6A‐C). In addition to cardiac apoptosis, cardiac ferroptosis was also found in rats with I/R injury as indicated by an increase in ACSL4 and a decrease in GPX4 protein levels, compared to sham‐operated rats (Figure 7A‐B). Caspase 8 is a molecular switch for the cells undergoing either apoptosis or necroptosis.^28^ Our results showed that cardiac I/R promoted cleaved caspase 8/Procaspase 8, p‐RIPK1/RIPK1 and p‐RIPK3/RIPK3 protein levels (Figure 7C‐E). However, p‐MLKL/MLKL, which is an executioner of necroptosis, was not affected by cardiac I/R injury, compared to sham‐operated rats (Figure 7F). These results indicated that it was apoptosis and ferroptosis, but not necroptosis, which occurred in the heart of rats with I/R injury.

### 3.2 Apoptosis and ferroptosis inhibitors, but not necroptosis inhibitor, reduced infarct size in rats with I/R injury

To investigate the roles of pharmacological interventions of different cell death pathways and their association with the infarct size reduction, inhibitors of apoptosis (Z‐vad), ferroptosis (Fer‐1) and necroptosis (Nec‐1) were used. For quantification analyses, data from Evans‐blue staining showed that the %AAR/LV area was no difference between groups (Figure 2B). In the determination of infarct size, our results demonstrated that %infarct size/AAR was significantly reduced in rats treated with low and medium doses of Z‐vad, and medium and high doses of Fer‐1, whereas other treatment regimens and treatment with Nec‐1 did not reduce the infarct size in rats with I/R injury (Figure 2C).

In cardiac I/R groups, all treatments affected neither arrhythmia score nor time to the first VT/VF onset, compared to the vehicle group (Figure 2D‐E). Data showed that QRS duration was prolonged in all rats with cardiac I/R injury, compared with the sham group and the treatments did not alter the QRS duration (Figure 2F). Moreover, QTc interval was not affected by this setting of cardiac I/R injury (Figure 2G). Cardiac gap junction function determined by the levels of Cx43 phosphorylation at serine‐368 residue and the ratio of p‐Cx43^ser368^/Cx43 was decreased in the vehicle group, compared to the sham group (Figure 2H). All pharmacological interventions did not affect this parameter, compared to the vehicle group (Figure 2H).

### 3.3 Apoptosis and ferroptosis inhibitors, but not necroptosis inhibitor, improved LV function in rats with I/R injury

The heart rate (HR) was increased during ischaemia, whereas treatment with low and medium doses of Z‐vad, and medium and high doses of Fer‐1 reduced heart rate during ischaemia, when compared to the vehicle group (Figure 3A). Other treatment regimens did not affect HR during ischaemia (Figure 3A). During reperfusion, HR was not different among groups (Figure 3A). Treatment with low and medium doses of Z‐vad, and medium and high doses of Fer‐1 increased %LVEF, SV, ±d*P*/d*t* and reduced LVEDP in both ischaemic and reperfusion periods, compared to the vehicle group (Figure 3B, C, E‐G). An increased LVESP was found in these treatment groups only in the reperfusion period, and not in the ischaemic period, compared to the vehicle group (Figure 3D). However, treatment with other regimens and Nec‐1 did not improve LV function in rats with I/R injury (Figure 3A‐G).

### 3.4 Apoptosis and ferroptosis inhibitors, but not necroptosis inhibitor, reduced cardiac mitochondrial dysfunction in rats with I/R injury

Treatment with low and medium doses of Z‐vad, and all doses of Fer‐1 effectively reduced mitochondrial ROS levels and prevented mitochondrial membrane depolarization (Figure 4A‐B). Furthermore, medium and high doses of Fer‐1 significantly decreased mitochondrial swelling in rats with I/R injury (Figure 4C). Treatment with high dose of Z‐vad, and all doses of Nec‐1 resulted in no measurable benefit on cardiac mitochondrial function following cardiac I/R injury (Figure 4A‐C). Representative pictures of cardiac mitochondria are shown in Figure 4D.

### 3.5 Ferroptosis inhibitor restored the balance of mitochondrial dynamics in rats with I/R injury

Treatment with all doses of Fer‐1 effectively suppressed cytosolic p‐Drp1^ser616^/Drp1 and enhanced Mfn2 protein levels, compared to the vehicle group (Figure 4E, H). Only treatment with a high dose of Fer‐1 decreased mitochondrial Drp1 and increased mitochondrial OPA1 protein levels in rats with I/R injury, compared to the vehicle group (Figure 4F, I). Treatment with Z‐vad and Nec‐1 did not reduce the imbalance of mitochondrial dynamics in rats with I/R injury (Figure 4E‐I). Mfn1 did not change during cardiac I/R and following all treatments, compared to sham‐operated rats (Figure 4G).

### 3.6 Apoptosis inhibitor promoted mitochondrial antioxidants, whereas necroptosis and ferroptosis inhibitors diminished cardiac inflammation in rats with I/R injury

The regulated cell death inhibitors had impact on the different pathways as our results demonstrated that all doses of Z‐vad led to increased SOD2 protein levels compared to the vehicle group (Figure 5A), while Nec‐1 and Fer‐1 treatments did not affect SOD2 protein levels at all. However, treatment with a high dose of Nec‐1 and all doses of Fer‐1 significantly reduced TNF‐α and p‐NF‐κB/NF‐κB protein levels, whereas low and medium doses of Nec‐1 and all doses of Z‐vad did not reduce these inflammatory markers, compared to the vehicle group (Figure 5B‐C).

### 3.7 Cardiac I/R injury led to cardiac apoptosis, which was reduced by apoptosis and ferroptosis inhibitors, but not necroptosis inhibitor

Treatment with all doses of Z‐vad effectively suppressed the Bax/Bcl‐2 ratio, Cyt *c* and cleaved caspase 3/Procaspase 3 protein levels, compared to the vehicle group (Figure 6A‐C). Furthermore, medium and high doses of Fer‐1 reduced Bax/Bcl‐2 protein levels, and all doses of Fer‐1 decreased Cyt *c* and cleaved caspase 3/Procaspase 3 protein levels, compared to the vehicle group (Figure 6A‐C). However, Nec‐1 did not reduce cardiac apoptosis in rats with I/R injury (Figure 6A‐C). Representative images of TUNEL positive cells are shown in Figure 6D.

### 3.8 Cardiac I/R injury led to cardiac ferroptosis, which was reduced by apoptosis and ferroptosis inhibitors, but not necroptosis inhibitor

Treatment with medium and high doses of Fer‐1 significantly decreased ACSL4 protein levels, and a high dose of Fer‐1 increased GPX4 protein levels, compared to the vehicle group (Figure 7A‐B). Treatment with low and medium doses of Z‐vad reduced ACSL4 protein levels, and GPX4 protein levels were increased in rats treated with a medium dose of Z‐vad (Figure 7A‐B). However, other treatment regimens and Nec‐1 did not reduce ferroptosis in rats with I/R injury (Figure 7A‐B).

### 3.9 Cardiac necroptosis was not involved in the pathogenesis of cardiac I/R injury

Treatment with low and medium doses of Nec‐1 reduced cleaved caspase 8/Procaspase 8 protein levels and all doses of Nec‐1 effectively suppressed p‐RIPK1/RIPK1 and p‐RIPK3/RIPK3 protein levels, compared to the vehicle group (Figure 7C‐E). Interestingly, all doses of Z‐vad also significantly decreased the levels of cleaved caspase 8/Procaspase 8, compared to the vehicle group (Figure 7C). In addition, medium and high doses of Fer‐1 also reduced p‐RIPK1/RIPK1 protein levels, compared to the vehicle group (Figure 7D). However, all treatment did not alter p‐MLKL/MLKL protein levels, compared to sham and vehicle groups (Figure 7F).

## 4 DISCUSSION

Findings from this study demonstrated that: (1) Myocardial infarction and LV dysfunction caused by cardiac I/R injury were mediated by cardiac apoptosis and ferroptosis, along with inflammation, oxidative stress, mitochondrial dysfunction and mitochondrial dynamic imbalance; (2) The apoptosis and ferroptosis inhibitors effectively reduced the infarct size and LV dysfunction in rats with I/R injury, in which the low and medium doses of apoptosis inhibitor, medium and high doses of ferroptosis inhibitor were the most effective doses; (3) The apoptosis inhibitor (at low and medium doses) prevented cardiac I/R injury by reducing apoptosis, ferroptosis, oxidative stress and mitochondrial dysfunction; (4) The ferroptosis inhibitor (at medium and high doses) showed cardioprotective benefits against cardiac I/R injury by decreasing ferroptosis, apoptosis, oxidative stress, inflammation, mitochondrial dysfunction and mitochondrial dynamic imbalance; (5) The necroptosis inhibitor attenuated cardiac inflammation, but this was insufficient to improve functional outcomes in rats with I/R injury.

Cardiomyocyte death contributes to myocardial infarction and LV dysfunction during cardiac I/R injury.^7^ It has been shown that reperfusion therapy can lead to cardiac cell death accompanied by excessive oxidative stress, inflammation, and mitochondrial dysfunction.^7^, ^29^ In this study, we have highlighted that apoptosis and ferroptosis were mainly involved in myocardial infarction and LV dysfunction during cardiac I/R injury. Thus, apoptosis and ferroptosis inhibitors offer possible therapeutic routes for targeting cardiac I/R injury.

Either apoptosis or ferroptosis has reportedly controlled the expansion of the infarct size after reperfusion, contributing to LV dysfunction.^30^, ^31^, ^32^ Additionally, an enhanced apoptosis accelerated post‐infarction cardiac remodelling after MI.^33^ Mechanistically, a large amount of oxidative stress, that is generated during reperfusion, disrupted mitochondrial membrane integrity, resulting in cytochrome c leakage to the cytosol.^33^, ^34^, ^35^ This step is critical for the activation of caspase 3 and chromatin condensation, thus produced the morphological and biochemical characteristics of apoptosis.^36^ Furthermore, reperfusion of the ischaemic myocardium enhanced the excessive peroxidation of arachidonic acid and diminished Gpx4 activity, leading to ferroptosis.^37^

Z‐vad is a pan‐caspase inhibitor, and our results showed that low and medium doses (1.65 mg/kg and 3.3 mg/kg) of Z‐vad effectively inhibited caspase 3 and 8 activities in rats with I/R injury, leading to infarct size reduction (approximately 40%) and improved LV function. Consistent with findings from previous studies, they showed that 1.5–3.3 mg/kg of Z‐vad limited infarct size and improved LV function following cardiac I/R injury.^17^, ^38^ Nevertheless, our data showed that the cardioprotective effects of Z‐vad disappeared when we increased the dose to 6.6 mg/kg, while caspase activity was still suppressed. The undesirable effect of high‐dose Z‐vad was also reported in the previous studies, and it was suggested that a high‐dose of Z‐vad paradoxically augmented cell death in TNF‐α‐treated neutrophils and in cancer cell lines.^39^, ^40^ Since these data suggested that caspase inhibition alone might not be sufficient to reduce cardiac damage caused by I/R injury, we further investigated other apoptosis‐related mechanisms including mitochondrial function and dynamics. Mitochondria are known as the centres of cellular metabolism and also that they can control cell death.^41^ Consistent with previous studies, this study showed that cardiac I/R injury led to mitochondrial dysfunction and mitochondrial dynamic imbalance.^22^, ^42^ We also found that mitochondrial superoxide levels were decreased following cardiac I/R injury. In this study, low and medium doses of Z‐vad effectively increased mitochondrial antioxidant levels, leading to decreased mitochondrial ROS levels, and mitochondrial membrane depolarization and subsequently reducing Cyt *c* release and apoptosis. However, mitochondrial swelling was not affected by Z‐vad treatment. Since mitochondrial permeability transition pores (mPTP) are the major route for water influx to the mitochondria during cardiac I/R injury,^43^ we speculated that Z‐vad could not reduce mPTP opening, and that this could be a possible explanation why Z‐vad did not reduce mitochondrial swelling in our study.

Excessive apoptosis has been considered as a part of pathological process of several cardiac diseases including MI.^44^ Various pharmacological interventions such as olmesartan, simvastatin, angiotensin converting enzyme inhibitor and β‐adrenergic receptor blockers suppressed cardiac apoptosis, evidenced by decreased caspase 3 activity and further improved cardiac function after MI.^33^ Some ER stress blockers such as salubrinal, EN460, QM295, 4‐Phenylbutynic acid and 17‐allylamino‐17‐demethoxy geldanamycin also inhibited cardiac I/R injury through a decreased apoptosis.^33^ On the other hand, etanercept, which is a TNF‐α receptor antagonist did not exert cardioprotective effect in MI patients, even though TNF‐α is an important activator of extrinsic apoptosis.^33^, ^45^ Collectively, data from our and others suggested that targeting apoptosis signaling, specifically caspases, provided cardioprotection against cardiac I/R injury better than an upstream of apoptosis pathway.

**FIGURE 8:**
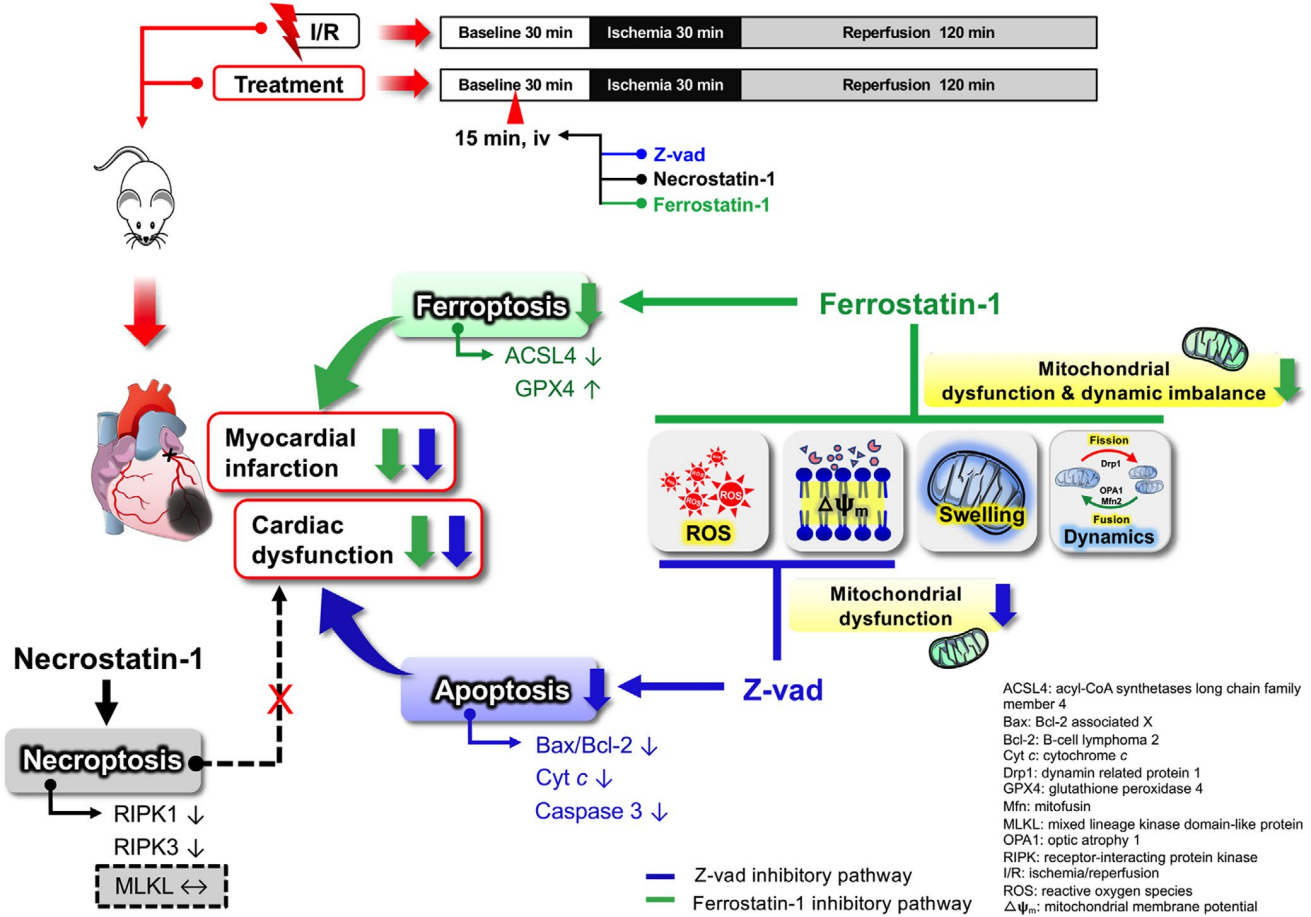
Proposed mechanisms of apoptosis inhibitor and ferroptosis inhibitor in mediating cardioprotection against cardiac I/R injury. ACSL4: Long‐chain‐fatty‐acid—CoA ligase 4; Cyt *c*: Cytochrome *c*; GPX4: Glutathione peroxidase 4; I/R: Ischaemia/reperfusion; MLKL: Mixed‐Lineage Kinase Domain‐Like Pseudokinase; RIPK: Receptor‐interacting protein kinases; ROS: reactive oxygen species

Fer‐1 is a potent ferroptosis inhibitor acting by targeting the lipid peroxidation process,^46^ and we demonstrated its promising therapeutic efficacy in reducing cardiac I/R injury. In this study, medium and high doses of Fer‐1 reduced ACSL4 and enhanced GPX4 protein levels, thereby reducing mitochondrial ROS production, mitochondrial membrane depolarization, mitochondrial swelling and mitochondrial dynamic imbalance. These beneficial effects also contributed to the reduction in infarct size (approximately 42%) and improved LV function during cardiac I/R injury. Intriguingly, the disappearance of mitochondrial cristae is one of the characteristics of ferroptosis.^47^ Data from this study indicate that mitochondrial swelling is predominantly regulated by the ferroptosis pathway, since this abnormality can only be prevented by this ferroptosis inhibitor. Moreover, mitochondrial dynamic imbalance was only decreased by the ferroptosis inhibitor, suggesting that this ferroptosis inhibitor had a higher potency in the prevention of mitochondrial impairment than did the apoptosis inhibitor. These findings are concordant with a previous study in which, in the main, ferroptosis was responsible for cardiac I/R injury in mice.^48^ Interestingly, Fer‐1 also markedly reduced the intrinsic apoptosis pathway in cardiac I/R injury through the inhibition of Cyt *c* release‐induced caspase 3 activation. The proposed mechanisms of apoptosis inhibitor and ferroptosis inhibitor in mediating cardioprotection against cardiac I/R injury is depicted in Figure 8.

Unlike apoptosis and ferroptosis, necroptosis was not observed in our cardiac I/R model (30‐min ischaemia and 2‐h reperfusion), and this finding was inconsistent with a previous study. In rats with cardiac I/R (30‐min to 1‐h ischaemia and 3–24 h or 4‐weeks reperfusion), MLKL protein expression was shown to be upregulated.^49^, ^50^, ^51^ These inconsistent findings suggested that the duration of the cardiac I/R protocol could markedly affect the contribution of regulated cell death pathways. If this hypothesis is true, our findings also strongly suggest that different necrotic cell death pathways could influence cardiac damage and cardiac contractile dysfunction differently depending on the duration of the ischaemia and reperfusion occurring in the heart, and future studies are needed to investigate this important issue and to warrant clinical translation of these findings.

In this study, our data demonstrated that apoptosis and ferroptosis are the major modes of cell death that responsible for cardiac I/R injury, whereas necroptosis is not participated in the pathogenesis of this acute cardiac I/R setting. To achieve a successful reperfusion therapy with a minimal adverse effect, treatment with apoptosis or ferroptosis inhibitor might be considered as a possible adjuvant therapy in acute ST‐elevation myocardial infarction (STEMI) patients who undergoing percutaneous intervention or other reperfusion therapy. However, this study has a limitation in which each cell death inhibitor was given to the rats as a pretreatment regimen that is not relevant to the clinical setting. Thus, future studies are needed to warrant the cardioprotective effect of apoptosis and ferroptosis inhibitor against cardiac I/R injury under the clinically relevant setting by administration of the inhibitors during either ischaemia or at the onset of reperfusion. Regarding to the arrhythmias, this is an acute study to test whether cell death inhibitors reduce arrhythmias during cardiac I/R injury; however, our results demonstrated that a single administration of cell death inhibitors did not change the arrhythmia score. This result might be due to a short detection of arrhythmia in our protocol. Nevertheless, in our I/R protocol Nec‐1 did not exert favourable functional outcomes since cardiac I/R, particularly in the setting of 30‐min ischaemia and 2‐h reperfusion, did not increase MLKL phosphorylation to instigate necroptosis, thus necroptosis did not occur, and Nec‐1 did not interfere with MLKL function.

## 5 CONCLUSION

Our study provides novel evidence that apoptosis and ferroptosis are the significant modes of cell death associated with acute cardiac I/R injury in rats. Apoptosis and ferroptosis inhibitors exerted cardioprotective effects against cardiac I/R injury through the modulation of mitochondrial function and the inhibition of apoptosis and ferroptosis pathways.

## CONFLICT OF INTEREST

The authors declare that they have no conflict of interest.

## AUTHOR CONTRIBUTIONs

**Ying Luo:** Data curation (equal); Formal analysis (equal); Investigation (equal); Methodology (equal); Writing – original draft (equal). **Nattayaporn Apaijai:** Data curation (lead); Formal analysis (lead); Funding acquisition (equal); Investigation (lead); Methodology (lead); Validation (lead); Visualization (equal); Writing – original draft (lead). **Suchan Liao:** Data curation (supporting); Formal analysis (supporting); Investigation (supporting); Methodology (supporting); Writing – original draft (supporting). **Chayodom Maneechote:** Formal analysis (supporting); Investigation (supporting); Methodology (supporting); Writing – original draft (supporting). **Titikorn Chunchai:** Data curation (supporting); Formal analysis (supporting); Investigation (supporting); Methodology (supporting); Writing – original draft (supporting). **Busarin Arunsak:** Data curation (supporting); Formal analysis (supporting); Investigation (supporting); Methodology (supporting); Writing – original draft (supporting). **Juthipong Benjanuwattra:** Formal analysis (supporting); Investigation (supporting); Methodology (equal); Visualization (equal); Writing – original draft (supporting). **Panat Yanpiset:** Data curation (supporting); Formal analysis (supporting); Writing – original draft (supporting). **Siriporn Chattipakorn:** Data curation (supporting); Funding acquisition (supporting); Investigation (supporting); Methodology (supporting); Writing – original draft (supporting). **Nipon Chattipakorn:** Conceptualization (lead); Funding acquisition (lead); Project administration (lead); Supervision (lead); Validation (lead); Visualization (equal); Writing – review & editing (lead).

## Data Availability

Original data of this manuscript are available on reasonable request to the corresponding author.
