# Corrigendum

**DOI:** 10.1111/jcmm.17705

**Published:** 2023-03-28

In Ying Luo et al.,^1^ the representative TUNEL pictures in Figure 6D are incorrect. The correct figures are shown below. The error affects none of the statistical analyses or conclusions, and the scientific content is unaltered.

**FIGURE 6:**
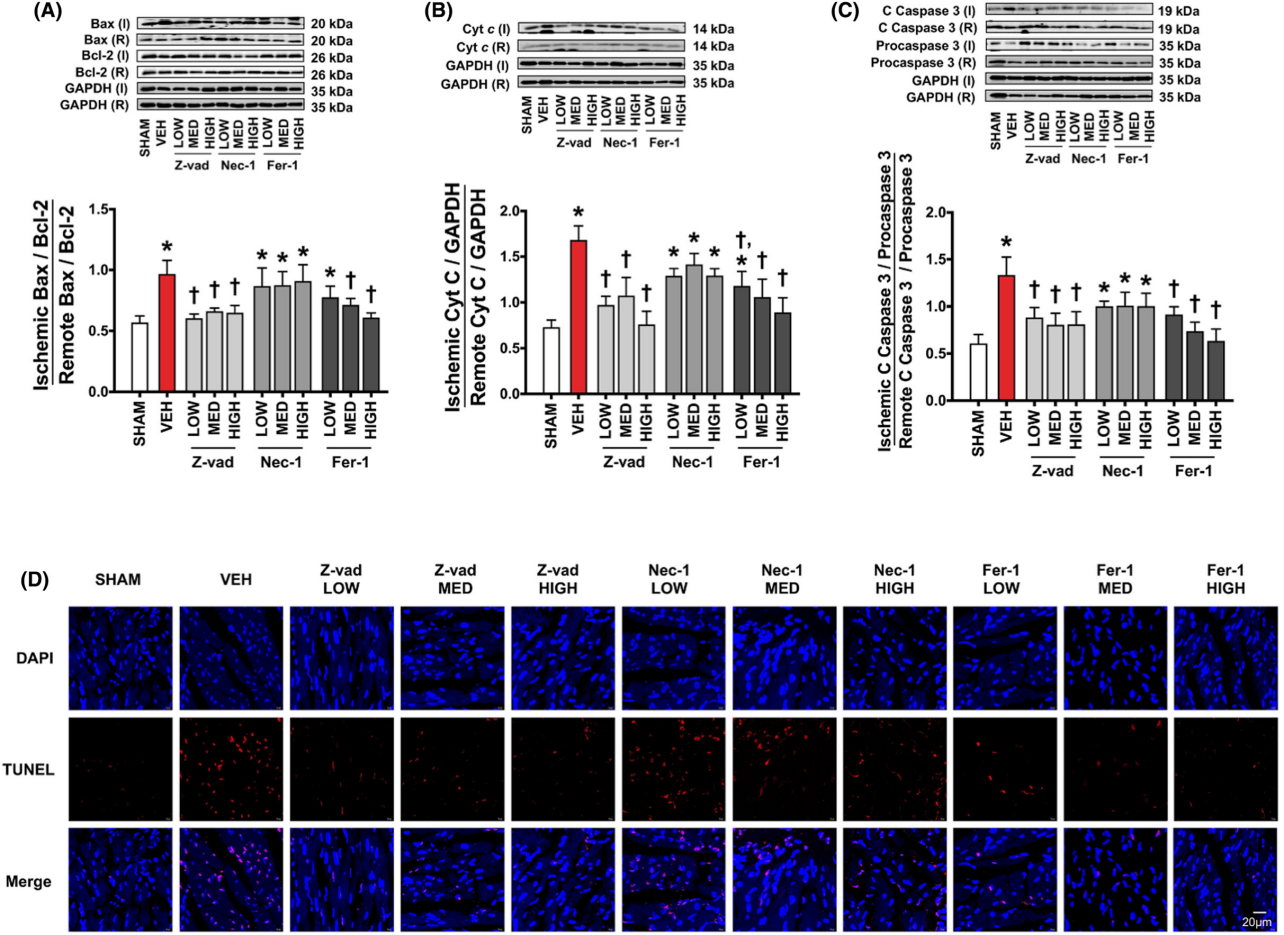
The effects of apoptosis, necroptosis, and ferroptosis inhibitors on cardiac apoptosis in rats with cardiac I/R injury (*n* = 6/group). (A) Bax/Bcl‐2, (B) Cyt c, (C) Cleaved caspase 3/Procaspase 3, (D) Representative TUNEL pictures (scale bar = 20 μm). Data are presented as means ± SEM. *p < 0.05 vs. sham, ^†^ *p* < 0.05 vs vehicle. Bax, Bcl‐2 Associated X; C, cleaved; Cyt c, cytochrome c; DAPI, 4′,6‐diamidino‐2‐phenylindole; Fer‐1, ferrostatin‐1; GAPDH, glyceraldehyde 3‐phosphate dehydrogenase; HIGH, high dose; I: ischemic; LOW, low dose; MED, medium dose; Nec‐1, necrostatin‐1; R, remote; TUNEL, Terminal deoxynucleotidyl transferase dUTP nick end labelling; VEH, vehicle; Z‐vad, Z‐vad.fmk.
